# Cognitive and Behavioral Features of Patients With Amyotrophic Lateral Sclerosis Who Are Carriers of the *TARDBP* Pathogenic Variant

**DOI:** 10.1212/WNL.0000000000208082

**Published:** 2024-01-23

**Authors:** Cristina Moglia, Andrea Calvo, Antonio Canosa, Umberto Manera, Rosario Vasta, Francesca Di Pede, Margherita Daviddi, Enrico Matteoni, Maura Brunetti, Luca Sbaiz, Sara Cabras, Salvatore Gallone, Maurizio Grassano, Laura Peotta, Francesca Palumbo, Gabriele Mora, Barbara Iazzolino, Adriano Chio

**Affiliations:** From the Rita Levi Montalcini' Department of Neuroscience (C.M., A. Calvo, A. Canosa, U.M., R.V., F.D.P., M.D., E.M., M.B., S.C., M.G., L.P., F.F.P., G.M., B.I., A. Chio), University of Torino; Neurology 1 (C.M., A. Calvo, A. Canosa, U.M., L.S., S.G., A. Chio), Azienda Ospedaliero-Universitaria Città della Salute e della Scienza of Torino; and Institute of Cognitive Sciences and Technologies (A. Canosa, A. Chio), National Research Council, Rome, Italy.

## Abstract

**Background and Objectives:**

*TARDBP* patients are considered particularly prone to cognitive involvement, but no systematic studies of cognitive impairment in *TARDBP* patients are available. The aim of this article was to depict in depth the cognitive-behavioral characteristics of a cohort of patients with amyotrophic lateral sclerosis (ALS) carrying *TARDBP* pathogenetic variants followed by an ALS referral center.

**Methods:**

We enrolled all patients with ALS seen at the Turin ALS expert center in the 2009–2021 period who underwent extensive genetic testing and a neuropsychological battery encompassing executive function, verbal memory, language, visual memory, visuoconstructive abilities, attention/working memory, psychomotor speed, nonverbal intelligence, cognitive flexibility, social cognition, and behavior. Tests were compared with the Mann-Whitney *U* test on age-corrected, sex-corrected, and education-corrected scores. Cognition was classified as normal (ALS-CN); isolated cognitive impairment (ALSci), that is, evidence of executive and/or language dysfunction; isolated behavioral impairment (ALSbi), that is, identification of apathy; cognitive and behavioral impairment (ALScbi), that is, evidence meeting the criteria for both ALSci and ALSbi; and frontotemporal dementia (ALS-FTD).

**Results:**

This study includes 33 patients with *TARDBP* pathogenetic variants (*TARDBP*-ALS) (median age 61 years [interquartile range (IQR) 53–67], 8 female [24.2%]) and 928 patients with ALS not carrying the pathogenic variant (WT-ALS) (median age 67 years [IQR 59–74], 386 female [41.6%]). TARDBP-ALS cases were also compared with 129 matched controls (median age 66 years [IQR 57.5–71.5], 55 female [42.6%]). TARDBP-ALS and WT-ALS patients were cognitively classified as ALS-CN (54% vs 58.8%, respectively), ALSci (21.2% vs 18.3%), ALSci (9.1% vs 9.5%), ALScbi (6.1% vs 6.0%), and ALS-FTD (9.1 vs 6.7%), with no significant difference (*p* = 0.623). Compared with controls, TARDBP-ALS had a worse performance in executive functions, visual memory, visuoconstructive abilities, verbal fluency, and the apathy behavioral component of FrSBe. The scores of performed tests, including all Edinburgh Cognitive and Behavioral ALS Screen subdomains, were similar in TARDBP-ALS and WT-ALS.

**Discussion:**

TARDBP-ALS patients were significantly more impaired than controls in most examined domains but do not show any specific pattern of cognitive impairment compared with WT-ALS. Our findings are relevant both clinically, considering the effect of cognitive impairment on patients' decision-making and caregivers' burden, and in designing clinical trials for the treatment of patients carrying *TARDBP* pathogenetic variants.

## Introduction

Amyotrophic lateral sclerosis (ALS) is a neurodegenerative disorder, characterized by the progressive impairment of upper and lower motor functions. Cognitive or behavioral impairment is diagnosed in up to half of patients with ALS, and in 15% of patients, the clinical criteria of frontotemporal dementia (FTD) are met.^1,2^ In approximately 10%–15% of patients, a pathogenetic variant of one of the ALS-related genes is identified, most commonly *C9orf72*, *SOD1*, *TARDBP*, and *FUS*.^3^ Among ALS genes, *C9orf72* has been reported to be related to specific cognitive-behavioral signatures.^4-7^ Conversely, the cognitive characteristics of other ALS-related genes, such as *TARDBP*, have been much less explored, and our understanding is mostly based on case reports and few case series with poor details on impaired cognitive domains.^8^ Generally, *TARDBP* patients are considered particularly prone to cognitive involvement, and *TARDBP* gene assessment is included in the genetic panel of patients with FTD.^9-11^ Therefore, the aim of this article was to depict in depth the cognitive-behavioral characteristics of a cohort of patients with ALS carrying pathogenetic of *TARDBP* genes followed by an ALS referral center, comparing them with controls and participants with ALS not carrying pathogenic variants (WT-ALS).

## Methods

This study includes all patients with ALS seen at the Turin ALS expert center in the 2009–2021 period and who underwent both cognitive/behavioral and extensive genetic testing. Patients were eligible if they had a diagnosis of ALS according to Gold Coast criteria.^12^ The ALS Functional Rating Scale–revised score was assessed at the time of cognitive testing. Patients with a history of disorders which may potentially affect cognition (i.e., major stroke, severe head injuries, and mental retardation), alcohol or drug dependence, severe mental illness, or use of high-dose psychoactive medications were tested but not included in data analysis. Patients who were not of Italian native language were assessed only through an unstructured interview and therefore were excluded from the analysis. The same cognitive and behavioral battery was administered to 129 age-matched and sex-matched participants.

### Neuropsychological Assessment

The neuropsychological battery was selected according to the Diagnostic Criteria for the Behavioral Variant of Frontotemporal Dementia,^13^ and ALS-FTD Consensus Criteria (ALSFTD-CC)^14^ and included tests encompassing executive function, verbal and visual memory, attention and working memory, visuospatial function, language, social cognition, and behavior. The tests were administered within 2 months from diagnosis.^2^ A detailed list of tests is reported in eMethods (links.lww.com/WNL/D363). According to the ALSFTD-CC,^14^ patients were classified in 5 categories: patients with normal cognition (ALS-CN); patients with isolated cognitive impairment (ALSci), that is, patients with evidence of executive and/or language dysfunction; patients with isolated behavioral impairment (ALSbi), that is, patients with apathy with or without other behavioral changes; patients with both cognitive and behavioral impairment (ALScbi), that is, patients meeting the criteria for both ALSci and ALSbi; and patients with frontotemporal dementia (ALS-FTD).

### Domain Classification of Tests

Tests were classified according to the main neuropsychological domain they assess (eTable 1, links.lww.com/WNL/D363).^14-17^ In detail, executive function was tested with Letter Fluency Test (FAS), Category Fluency Test (CAT), Trail Making Test B-A (TMT B-A), Frontal Assessment Battery (FAB), Edinburgh Cognitive and Behavioral ALS Screen (ECAS) Executive Function score, and ECAS Verbal Fluency score; verbal memory with Rey Auditory Verbal Learning Test–Immediate Recall, Rey Auditory Verbal Learning Test–Delayed Recall, Babcock Story Recall Test–Immediate Recall, Babcock Story Recall Test–Delayed Recall, and ECAS Memory score; language was assessed with Token test and semantic system tests (7 and 8) of the Battery for the Analysis of Aphasic Deficits before 2016 and with ECAS Language score and Boston Naming Test from 2016; visual memory with Rey-Osterrieth Complex Figure Test–Differed Recall (ROCF-DR); visuoconstructive abilities with Rey-Osterrieth Complex Figure Test–Immediate Recall (ROCF-IR), Clock Drawing Test (Clock), and ECAS Visuospatial Abilities score; attention/working memory with Digit Span Forward (FW) and Digit Span Backward (BW); psychomotor speed with Trail Making Test A (TMT A); nonverbal intelligence with Raven Colored Progressive Matrices (CPM47); cognitive flexibility with Trail Making Test B (TMT B); and theory of mind (social cognition) with Story-Based Empathy Task (SET).

Each domain was considered impaired if at least one of the tests had a score under the normative cutoff, with the exception of executive functions and verbal memory, which were considered impaired if at least two not overlapping tests had a score under the cutoff.^14^

### Genetic Screening

A total of 1,159 patients did cognitive testing. Among them, 957 underwent whole-genome sequencing (WGS). WGS methodology and quality control filters are reported in detail elsewhere.^18^ These patients were those for whom we had enough DNA when we performed WGS. Pathogenetic variants of 46 ALS-related genes were extracted.^19^ Variant classification was based on the 2015 American College of Medical Genetics and Genomics—Association for Molecular Pathology guidelines.^20^ For the remaining 202 patients, all the coding exons and 50 bp of the flanking intron-exon boundaries of *SOD1*, of exon 6 of *TARDBP*, and of exons 14 and 15 of *FUS* have been PCR amplified, sequenced using the Big-Dye Terminator v3.1 sequencing kit (Applied Biosystems Inc., Waltham, MA), and run on an ABIPrism 3130 genetic analyzer. We selected these exons as the vast majority of known pathogenic variants that are known to lie within these mutational hotspots. A repeat-primed PCR assay was used to screen for the presence of the GGGGCC hexanucleotide expansion in the first intron of *C9ORF72*.^21^ Repeat lengths of ≥30 units with the characteristic sawtooth pattern were considered to be pathogenic.^21^

### Statistical Methods

Comparisons between tests were performed on age-corrected, sex-corrected, and education-corrected scores, using the most recent Italian normative.^6^ Since some cognitive tests scores had not a normal distribution, the Mann-Whitney *U* test was used for comparisons. Two-tailed *p* values are reported; Holmes correction for multiple testing was used. Cohen effect sizes were also calculated. All analyses were performed with SPSS 26.0 statistical package (SPSS, Chicago, IL).

### Standard Protocol Approvals, Registrations, and Patient Consents

The study was approved by the Ethics Committees of the ALS Expert Center of Torino (Comitato Etico Azienda Ospedaliero-Universitaria Città della Salute e della Scienza, Torino, #0038876). Patients and controls provided written informed consent before enrollment. The databases were anonymized according to the Italian law for the protection of privacy.

### Data Availability

Data will be available on reasonable request by interested researchers.

## Results

**Figure 1:**
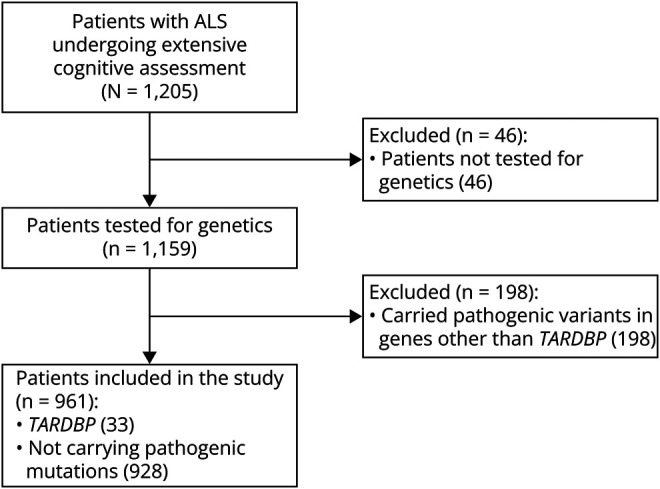
Flowchart Reporting the Patients' Selection Process ALS = amyotrophic lateral sclerosis.

In the period 2009–2021, a total of 1,205 patients with ALS seen at the Turin ALS center were tested for cognitive/behavioral function. In 46 of them, genetics was not performed. Of the remaining 1,159 patients, we excluded 198 cases because they carried pathogenic variants in genes other than *TARDBP* (eTable 2, links.lww.com/WNL/D363). Therefore, this study includes 33 patients with *TARDBP* pathogenetic variants (*TARDBP*-ALS) and 928 ALS patients with no genetic variants. A flow chart reporting the selection of patients is shown in Figure 1. The list of TARDBP-ALS cases is reported in eTable 3. TARDBP-ALS cases were apparently unrelated, although 14 of them were of Sardinian ancestry.

The cognitive function of TARDBP-ALS cases was also compared with that of 129 matched controls. The demographic and clinical characteristics of patients and controls are reported in eTable 4 (links.lww.com/WNL/D363). The median age at onset of controls (67 years [interquartile range (IQR) 59–74]) was significantly higher than that of TARDBP-ALS cases (61 years [IQR 53–67]) (*p* = 0.002), but their education level was similar (controls 10 years [IQR 8–13], TARDBP-ALS 11 years [IQR 8–13], *p* = 0.609). In addition, as expected due to the different penetrance of TARDBP pathogenic variants in the two sexes,^22^ female patients were underrepresented in the TARDBP-ALS cohort (8, 24.2%) compared with controls (55, 42.6%).

In Table 1, the clinical and demographic characteristics of TARDBP-ALS patients are compared with those of WT-ALS. The median age at onset of TARDBP-ALS was significantly lower than in WT-ALS (*p* < 0.0001 for both comparisons). The male frequency was significantly higher in TARDBP-ALS patients compared with WT-ALS (*p* = 0.046).

**Table 1:** Comparison of Demographic and Clinical Characteristics of *TARDBP* Cases and WT-ALS

	TARDBP-ALS (N = 33)	WT-ALS (N = 928)	*p* Value
Age at diagnosis, median (IQR)	61 (53–67)	67 (59–74)	0.002
Sex (female), n (%)	8 (24.2)	386 (41.6)	0.046
No. of education years, median (IQR)	11 (8–13)	8 (7–13)	0.201
Site of onset (spinal), n (%)	20 (60.6)	615 (67.9)	0.499
ALSFRS-R score at the time of neuropsychological tests, median (IQR)	42 (37–45)	40 (36–44)	0.174

Abbreviations: ALSFRS-R = amyotrophic lateral sclerosis functional rating scale–revised; IQR = interquartile range; WT = ALS patients not carrying pathogenic variants.

### Cognitive Status of TARDBP-ALS Patients

Of the 33 *TARDBP* patients, 18 (54.5%) had normal cognitive function, 7 (21.2%) ALSci, 3 (9.1%) ALSbi, 2 ALScbi (6.1%), and 3 (9.1%) ALS-FTD (Table 2). The overall frequency of cognitive impairment (ALSci/ALSbi/ALScbi/ALS-FTD) was not different between TARDBP-ALS and WT-ALS patients (15 [45.5%] vs 382 [42.1%], *p* = 0.623). Among TARDBP-ALS patients, no sex differences were found in cognitive function and the median age at onset of patients with normal cognitive function was lower than of those with ALSci/ALSbi/ALScbi (*p* = 0.036). There was no obvious correlation between the pathogenetic variants and cognitive status, although notably all the 3 patients with ALS-FTD carried the p.Ala382Thr variant. The proportion of cognitive impairment was identical in TARDBP-ALS patients with known family history for ALS or FTD (5/11, 45.5%) and in those with no known family history (10/22, 45.5%).

**Table 2:** Frequency of Cognitive Impairment in *TARDBP* and WT-ALS Patients

	*TARDBP* (N = 33)	WT-ALS (N = 928)
ALS-CN, n (%)	18 (54.5)	546 (58.8)
Cognitively impaired ALS, n (%)	15 (45.5)	382 (41.1)
ALSci, n (%)	7 (21.2)	170 (18.3)
ALSbi, n (%)	3 (9.1)	88 (9.5)
ALScbi, n (%)	2 (6.1)	62 (6.7)
ALS-FTD, n (%)	3 (9.1)	62 (6.7)

Abbreviations: ALS = amyotrophic lateral sclerosis; ALSbi = patients with isolated behavioral impairment; ALScbi = patients with both cognitive and behavioral impairment; ALSci = patients with isolated cognitive impairment; ALS-CN = patients with normal cognition; ALS FTD = patients with frontotemporal dementia; WT-ALS = ALS patients not carrying pathogenic variants.

ALS-CN vs cognitively impaired ALS, *p* = 0.623.

### Impaired Cognitive Tests and Domains in TARDBP-ALS Compared With Controls

Compared with controls, TARDBP-ALS had a worse performance in all tests assessing executive functions (FAS [*p* = 0.012], TMT B-A [*p* = 0.002], FAB [*p* = 0.0001], ROCF-IR [*p* < 0.0001], and Clock [*p* < 0.0001]) and in those assessing visual memory (ROCF-DR [*p* < 0.0001]), attention and working memory (Digit Span FW [*p* < 0.0001] and Digit Span BW [*p* = 0.001]), nonverbal general intelligence (CPM47 [*p* < 0.0001]), and cognitive flexibility (TMT B [*p* = 0.005]) (eTables 5 and 6, links.lww.com/WNL/D363). All SET test subscores were lower in TARDBP-ALS patients than in controls. These findings were also reflected by the higher impairment in two ECAS domains (executive [*p* = 0.014] and visuospatial [*p* = 0.034]). In addition, the Mini Mental State Examination score was significantly lower in TARDBP-ALS patients (*p* = 0.04). Hospital Anxiety and Depression Scale-Anxiety and Hospital Anxiety and Depression Scale-Depression scores were similar in TARDBP-ALS patients and controls.

### Comparison of Cognitive and Behavioral Impairment of TARDBP-ALS With WT-ALS Patients

**Figure 2:**
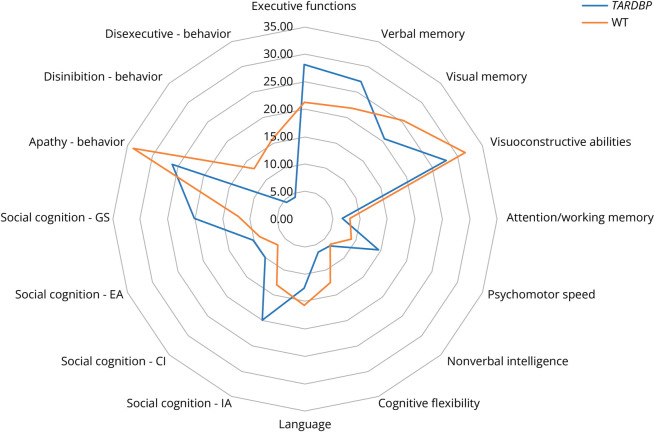
Radar Chart Comparing the Frequency of Impaired Cognitive and Behavioral Domains in Patients With *TARDBP* Pathogenetic Variants and WT-ALS No significant differences were detected. ALS = amyotrophic lateral sclerosis; WT-ALS = ALS patients not carrying pathogenic variants.

The scores of performed tests, including all ECAS subdomains, were similar in TARDBP-ALS and WT-ALS patients (Tables 3 and 4). In addition, anxiety and depression scores did not show differences. Since the mean age at onset and the gender distribution of TARDBP-ALS and WT-ALS were different, an exploratory evaluation was also performed comparing the 33 TARDBP-ALS patients with a randomly selected group of 99 WT-ALS matched by age and sex. No significant differences were found in the median scores of all tests, including ENCALS subdomains (eTables 6 and 7, links.lww.com/WNL/D363). At cognitive and behavioral domain levels, there were no significant differences between TARDBP-ALS and WT-ALS (Figure 2).

**Table 3:** Comparison of Cognitive Tests Between All ALS Patients With *TARDBP* Pathogenic Variants and WT-ALS

	TARDBP-ALS (N = 33)	WT-ALS (N = 928)	*p* Value	Effect size
MMSE	27.5 (25.9–30.0) N = 32	27.7 (26.3–30.0) N = 927	0.827	0.01
FAS	29.2 (18.8–37.2) N = 32	29.5 (22.7–36.7) N = 890	0.794	0.07
CAT	21.4 (14.6–24.9) N = 31	19.3 (15.8–22.3) N = 890	0.470	0.10
FAB	14.1 (12.0–16.4) N = 28	15.2 (13.5–16.7) N = 837	0.229	0.08
Digit Span FW	5.7 (4.9–6.5) N = 29	5.7 (5.0–6.4) N = 842	0.896	0.01
Digit Span BW	4.1 (3.2–4.7) N = 28	3.9 (3.4–4.5) N = 756	0.559	0.05
TMT A	36 (26–60) N = 31	37 (26–55) N = 820	0.844	0.04
TMT B	98 (71–175) N = 31	78 (41–146) N = 820	0.239	0.03
TMT B-A	59 (10–114) N = 31	41 (14–97) N = 820	0.437	0.05
RAVL-IR	38.6 (32.7–44.4) N = 21	39.7 (34.0–47) N = 602	0.661	0.11
RAVL-DR	7.0 (5.0–10.0) N = 358	7.0 (4.0–10.0) N = 602	0.612	0.13
BSRT-IR	6.5 (3.5–7.7) N = 14	5.9 (4.6–7.0) N = 500	0.825	0
BSRT-DR	6.2 (5.0–8.0) N = 14	6.7 (5.2–7.9) N = 500	0.744	0.16
ROCF-IR	31.4 (27.2–33.3) N = 29	31.5 (28.0–34.0) N = 705	0.921	0.02
ROCF-DR	11.0 (8.5–14.3) N = 29	12.0 (8.3–16.5) N = 705	0.343	0.04
Clock	5 (3–5) N = 28	4 (3–5) N = 813	0.33	0.18
CPM47	27.4 (23.2–30.2) N = 29	29.0 (24.8–31.6) N = 875	0.135	0.22
SET Intention Attribution	4.53 (2.92–5.25) N = 10	4.9 (3.9–6.0) N = 274	0.344	0.12
SET Causal Inference	4.86 (3.29–5.09) N = 10	4.9 (3.9–5.3) N = 274	0.567	0.03
SET Emotion Attribution	4.51 (3.81–5.4) N = 10	4.9 (3.3–6.0) N = 274	0.717	0.08
SET Global Score	13.9 (10.27–15.24) N = 10	14 (10.4–16) N = 274	0.535	0.05
HADS-A	5.5 (4–9.5) N = 22	7 (5–10) N = 681	0.335	0.20
HADS-D	3.5 (2.3–6.8) N = 22	5 (3–8) N = 681	0.306	0.31

Abbreviations: ALS = amyotrophic lateral sclerosis; BSRT = Babcock Story Recall Test; BW = backward; CAT = Category Fluency Test; Clock = Clock Drawing Test; CPM47 = Raven Colored Progressive Matrices; FAB = Frontal Assessment Battery; DR = delayed recall; FAS = Letter Fluency test; FW = forward; HADS-A = Hospital Anxiety and Depression Scale-Anxiety; HADS-D = Hospital Anxiety and Depression Scale-Depression; IR = Immediate Recall; MMSE = Mini Mental State Examination; RAVL = Rey Auditory Verbal Learning Test; ROCF = Rey-Osterrieth Complex Figure Test; SET = Story-Based Empathy Task; TMT = Trail Making Test; WT-ALS = ALS patients not carrying pathogenic variants.

Median values and interquartile ranges are reported. Cohen effect sizes are shown.

**Table 4:** Comparison of the Results of ECAS Between TARDBP-ALS and WT-ALS

	TARDBP-ALS (N = 16)	WT-ALS (N = 467)	*p* Value	Effect size
ECAS Language	25 (22–28)	26 (23–27)	0.900	0.06
ECAS Verbal Fluency	16 (8–16)	16 (12–20)	0.080	0.30
ECAS Executive	25 (22–39)	33 (25–38)	0.346	0.25
ECAS Memory	16 (12–20)	17 (14–20)	0.533	0.22
ECAS Visuospatial	12 (11–12)	12 (11–12)	0.971	0.08
ECAS ALS-specific	66 (50–83)	74 (61–82)	0.239	0.29
ECAS ALS nonspecific	28 (23–32)	29 (25–31)	0.650	0.12
ECAS total score	86 (77–111)	102 (88–113)	0.293	0.19

Abbreviations: ALS = amyotrophic lateral sclerosis; ECAS = Edinburgh Cognitive and Behavioral ALS Screen; WT-ALS = ALS patients not carrying pathogenic variants.

Median values and interquartile ranges are reported. Cohen effect sizes are also shown.

## Discussion

In this large cohort of patients with ALS, fully characterized for genetics and assessed with a comprehensive battery of tests, we found that 15 (45.5%) of the 33 TARDBP-ALS patients had a cognitive and/or behavioral impairment at the time of diagnosis, with a frequency slightly higher than in WT-ALS patients (382 of 928 cases, 41.1%). Regarding the specific cognitive and behavioral tests, we found that compared with controls, TARDBP-ALS patients were significantly more impaired in tests assessing executive functions, visual memory, visuoconstructive abilities, verbal fluency, and the apathy behavioral component of FrSBe; however, no significant differences were found between TARDBP-ALS and WT-ALS.

There are no reports evaluating in depth cognitive and behavioral impairment of TARDBP-ALS, although the cognitive classification of TARDBP-ALS patients has been reported in some series. In cohorts of TARDBP-ALS patients of Italian ancestry, 5.5% to 30.3% of patients had various degrees of cognitive impairment,^22-24^ but no detailed information related to impaired domains/tests was reported. Outside Italy, in a French cohort of 28 TARDBP-ALS, cognitive impairment was reported in two patients (7.1%), who carried the p.Gly295Ser and p.Gly384Arg variants,^25^ whereas no one of the 4 TARDBP-ALS patients reported in a study from Switzerland had cognitive impairment.^26^ Finally, a review paper on TARDBP-ALS patients of Chinese ancestry reported that cognitive impairment, expressed as full-blown FTD, is relatively rare in that population.^27^

It is of interest that the presence of *TARDBP* variants has been searched for in some series of FTD patients who underwent extensive genetic analysis (eTable 8, links.lww.com/WNL/D363). Overall, *TARDBP* pathogenetic variants in these FTD cohorts were quite rare (ranging from 0% to 7.7%). The two most common *TARDBP* variants reported to be associated with FTD are p.Ala382Thr and p.Ile383Val^9,11,28-30^; a few of these patients also presented motor signs at the time of cognitive evaluation. Notably, all our patients with ALS-FTD carried the p.Ala382Thr variant.

There are very few neuroimaging studies in TARDBP-ALS patients. An MRI study performed on 11 TARDBP-ALS patients (4 of whom with a diagnosis of ALSci) showed a distinctive reduction of gray matter volumes in the left precuneus and right angular gyrus; these brain areas are involved in language, number processing, spatial cognition, memory retrieval, attention, and theory of mind.^31^ A study with ^18^F-FDG-PET assessing 15 patients carrying the p.Ala382Thr variant found significant relative hypometabolism in TARDBP-ALS compared with WT-ALS in the right precentral and postcentral gyrus, superior and middle temporal gyrus, and insula, areas involved in social cognition, emotion, and social decision-making.^32^ Notably, 5 of 15 patients reported in that study are included in the present cohort. Taken together, MRI and PET studies confirm that in TARDBP-ALS patients, there is an involvement of cerebral areas related to cognitive function.

This study is not without limitations. First, cognitive and behavioral tests were performed at the time of diagnosis, thus reflecting the cognitive status in the early/intermediate phases of the disease. Second, ECAS was performed in patients seen from 2016. However, the number of patients evaluated with these tests is fairly representative of the whole cohort. Third, the performance in some neuropsychological tests used in this study may be negatively affected by patients' motor or bulbar impairment (namely, FAS, CAT, and TMT) and may have affected on the difference found between *TARBDP* cases and controls.

In conclusion, we showed that (1) TARDBP-ALS patients had worse cognitive and behavioral performances compared with matched controls in almost all examined tests/domains and in addition showed higher levels of anxiety and depression; (2) approximately 36% of TARDBP-ALS were diagnosed as ALSci/ALSbi or ALScbi, and 9.0%, all carrying the p.Ala382Thr pathogenic variant, had FTD, with a frequency similar to WT-ALS; and (3) TARDBP-ALS patients did not show any specific pattern of cognitive impairment compared with WT-ALS.

Our findings may have implications both in the clinical and research setting. Clinically, the high frequency of cognitive impairment in TARDBP-ALS could negatively affect patients' decision-making^33^ and increase caregivers' burden.^34^ In addition, the design of clinical trials specifically aimed at the treatment of patients carrying TARDBP-ALS pathogenic variants should consider the presence of cognitive and behavioral impairment of these patients.

## Appendix Authors

**Table:**

Name	Location	Contribution
Cristina Moglia, MD, PhD	Rita Levi Montalcini' Department of Neuroscience, University of Torino; Neurology 1, Azienda Ospedaliero-Universitaria Città della Salute e della Scienza of Torino, Turin, Italy	Drafting/revision of the manuscript for content, including medical writing for content; major role in the acquisition of data; study concept or design; analysis or interpretation of data; additional contributions: study supervision
Andrea Calvo, MD, PhD	Rita Levi Montalcini' Department of Neuroscience, University of Torino; Neurology 1, Azienda Ospedaliero-Universitaria Città della Salute e della Scienza of Torino, Turin, Italy	Drafting/revision of the manuscript for content, including medical writing for content; major role in the acquisition of data; study concept or design; analysis or interpretation of data; additional contributions: study supervision
Antonio Canosa, MD, PhD	Rita Levi Montalcini' Department of Neuroscience, University of Torino; Neurology 1, Azienda Ospedaliero-Universitaria Città della Salute e della Scienza of Torino, Turin; Institute of Cognitive Sciences and Technologies, National Research Council, Rome, Italy	Drafting/revision of the manuscript for content, including medical writing for content; analysis or interpretation of data
Umberto Manera, MD, PhD	Rita Levi Montalcini' Department of Neuroscience, University of Torino; Neurology 1, Azienda Ospedaliero-Universitaria Città della Salute e della Scienza of Torino, Turin, Italy	Drafting/revision of the manuscript for content, including medical writing for content; analysis or interpretation of data
Rosario Vasta, MD, PhD	Rita Levi Montalcini' Department of Neuroscience, University of Torino, Italy	Drafting/revision of the manuscript for content, including medical writing for content; analysis or interpretation of data
Francesca Di Pede, MD	Rita Levi Montalcini' Department of Neuroscience, University of Torino, Italy	Drafting/revision of the manuscript for content, including medical writing for content; major role in the acquisition of data; study concept or design
Margherita Daviddi, MD	Rita Levi Montalcini' Department of Neuroscience, University of Torino, Italy	Drafting/revision of the manuscript for content, including medical writing for content; major role in the acquisition of data; study concept or design; additional contributions: administrative, technical, or material support
Enrico Matteoni, MD	Rita Levi Montalcini' Department of Neuroscience, University of Torino, Italy	Drafting/revision of the manuscript for content, including medical writing for content; major role in the acquisition of data; study concept or design
Maura Brunetti, BSc	Rita Levi Montalcini' Department of Neuroscience, University of Torino, Italy	Drafting/revision of the manuscript for content, including medical writing for content; major role in the acquisition of data; study concept or design; additional contributions: administrative, technical, or material support
Luca Sbaiz, BSc	Neurology 1, Azienda Ospedaliero-Universitaria Città della Salute e della Scienza of Torino, Turin, Italy	Drafting/revision of the manuscript for content, including medical writing for content; major role in the acquisition of data
Sara Cabras, MD	Rita Levi Montalcini' Department of Neuroscience, University of Torino, Italy	Drafting/revision of the manuscript for content, including medical writing for content; major role in the acquisition of data; study concept or design
Salvatore Gallone, MD	Neurology 1, Azienda Ospedaliero-Universitaria Città della Salute e della Scienza of Torino, Turin, Italy	Drafting/revision of the manuscript for content, including medical writing for content; major role in the acquisition of data; study concept or design; additional contributions: administrative, technical, or material support
Maurizio Grassano, MD, PhD	Rita Levi Montalcini' Department of Neuroscience, University of Torino, Italy	Drafting/revision of the manuscript for content, including medical writing for content; major role in the acquisition of data; study concept or design; analysis or interpretation of data; additional contributions: administrative, technical, or material support
Laura Peotta, PsyD	Rita Levi Montalcini' Department of Neuroscience, University of Torino, Italy	Drafting/revision of the manuscript for content, including medical writing for content; major role in the acquisition of data; study concept or design; additional contributions: administrative, technical, or material support
Francesca Palumbo, MD	Rita Levi Montalcini' Department of Neuroscience, University of Torino, Italy	Drafting/revision of the manuscript for content, including medical writing for content; major role in the acquisition of data; study concept or design; additional contributions: administrative, technical, or material support
Gabriele Mora, MD	Rita Levi Montalcini' Department of Neuroscience, University of Torino, Italy	Drafting/revision of the manuscript for content, including medical writing for content; major role in the acquisition of data; study concept or design; analysis or interpretation of data; additional contributions: study supervision
Barbara Iazzolino, PsyD	Rita Levi Montalcini' Department of Neuroscience, University of Torino, Italy	Drafting/revision of the manuscript for content, including medical writing for content; major role in the acquisition of data; study concept or design; analysis or interpretation of data; additional contributions: study supervision
Adriano Chio, MD	Rita Levi Montalcini' Department of Neuroscience, University of Torino; Neurology 1, Azienda Ospedaliero-Universitaria Città della Salute e della Scienza of Torino, Turin; Institute of Cognitive Sciences and Technologies, National Research Council, Rome, Italy	Drafting/revision of the manuscript for content, including medical writing for content; major role in the acquisition of data; study concept or design; analysis or interpretation of data; additional contributions: obtained funding, study supervision

## Glossary

ALS: amyotrophic lateral sclerosis
ALSbi: patients with isolated behavioral impairment
ALScbi: patients with both cognitive and behavioral impairment
ALSci: patients with isolated cognitive impairment
ALS-CN: patients with normal cognition
ALS FTD: patients with frontotemporal dementia
ALSFTD-CC: ALS-FTD Consensus Criteria
BW: backward
CAT: Category Fluency Test
Clock: Clock Drawing Test
CPM47: Raven Colored Progressive Matrices
ECAS: Edinburgh Cognitive and Behavioral ALS Screen
FAB: Frontal Assessment Battery
FAS: Letter Fluency test
FW: forward
IQR: interquartile range
ROCF-DR: Rey-Osterrieth Complex Figure Test, differed recall
ROCF-IR: ROCF, Immediate Recall
SET: Story-Based Empathy Task
TMT: Trail Making Test
WGS: whole-genome sequencing
WT: ALS patients not carrying pathogenic variants
